# Severe Acute Respiratory Syndrome Coronavirus 2 and Pregnancy Outcomes According to Gestational Age at Time of Infection

**DOI:** 10.3201/eid2710.211394

**Published:** 2021-10

**Authors:** Dominique A. Badr, Olivier Picone, Elisa Bevilacqua, Andrew Carlin, Federica Meli, Jeanne Sibiude, Jérémie Mattern, Jean-François Fils, Laurent Mandelbrot, Antonio Lanzone, Danièle De Luca, Jacques C. Jani, Alexandre J. Vivanti

**Affiliations:** University Hospital Brugmann, Brussels, Belgium (D.A. Badr, A. Carlin, J.C. Jani);; Université Libre de Bruxelles, Brussels (D.A. Badr, A. Carlin, J.C. Jani);; Louis Mourier Hospital, AP-HP, Colombes, France (O. Picone, J. Sibuide, L. Mandelbrot);; Université de Paris, Paris, France (O. Picone, J. Sibuide, L. Mandelbrot);; Inserm IAME-U1137, Paris (O. Picone, J. Sibuide, L. Mandelbrot);; Groupe de Recherche sur les Infections pendant la Grossesse (GRIG), Velizy, France (O. Picone, J. Sibuide, L. Mandelbrot, A.J. Vivanti);; Gemelli University Polyclinic Foundation, Rome, Italy (E. Bevilacqua, F. Melli, A. Lanzone);; Antoine Béclère Hospital, AP-HP, Clamart, France (J. Mattern, A.J. Vivanti, D. De Luca);; Paris Saclay University, Paris (J. Mattern, D. De Luca, A.J. Vivanti);; Ars Statistica, Nivelles, Belgium (J.-F. Fils);; Catholic University Sacro Cuore, Rome (A. Lanzone)

**Keywords:** COVID-19, coronavirus disease, SARS-CoV-2, severe acute respiratory syndrome coronavirus 2, viruses, respiratory infections, zoonoses, high-risk pregnancy, pregnancy outcomes, propensity score matching, vaccination, Europe, PregOuTCOV

## Abstract

We conducted an international multicenter retrospective cohort study, PregOuTCOV, to examine the effect of gestational age at time of infection with severe acute respiratory syndrome coronavirus 2 (SARS-CoV-2) on obstetric and neonatal outcomes. We included all singleton pregnancies with a live fetus at 10 weeks’ gestation in which pregnancy outcomes were known. The exposed group consisted of patients infected with SARS-CoV-2, whereas the unexposed group consisted of all remaining patients during the same period. Primary outcomes were defined as composite adverse obstetric outcomes and composite adverse neonatal outcomes. Of 10,925 pregnant women, 393 (3.60%) were infected with SARS-CoV-2 (exposed group). After matching for possible confounders, we identified statistically significant increases in the exposed group of composite adverse obstetric outcomes at >20 weeks’ gestation and of composite adverse neonatal outcomes at >26 weeks’ gestation (p<0.001). Vaccination programs should target women early in pregnancy or before conception, if possible.

In early 2020, a new coronavirus, called severe acute respiratory syndrome coronavirus 2 (SARS-CoV-2), arrived in Europe. It infected millions of persons and led to the deaths of thousands by coronavirus disease (COVID-19) by May 2020, when numbers of infections per week in Europe decreased substantially. However, after a summer respite, the number of infections began to escalate again in September 2020, and several new variants were reported (*1*,*2*). Hundreds of articles published during this period reported the virus’s relationship with and effect on pregnancy and attempted to determine adverse neonatal and obstetric outcomes after infection. Meanwhile, mother-to-child transmission of SARS-CoV-2 has been established, and the World Health Organization recognized the virus as part of the TORCH (toxoplasmosis, other viruses, rubella, cytomegalovirus, and herpes simplex) family of infections (of which Zika virus was the most recent new member) (*3*,*4*), adding yet more interest to the possible perinatal consequences of SARS-CoV-2.

In a cohort study using propensity score-matching at the level of age, body mass index (BMI), and underlying conditions (e.g., diabetes, hypertension, asthma), Badr et al. demonstrated that pregnant women at >20 weeks of gestation (WG) infected with SARS-CoV-2 had a significantly higher risk for intensive care unit admission, endotracheal intubation, hospitalization for disease-related symptoms, and need for oxygen therapy (*5*). A systematic review demonstrated an increased risk for intensive care unit admission in infected pregnant women compared with infected nonpregnant women and noninfected pregnant women (*6*).

Many researchers have focused on the obstetric and neonatal outcomes of infected pregnant women. Some reports have demonstrated that rates of preterm and cesarean delivery have increased (*6*–*10*), whereas others have reported a close association between SARS-CoV-2 infection and preeclampsia or preeclampsia-like syndromes (*11*). Enormous effort has been made to learn more about adverse outcomes related to SARS-CoV-2 infection, but most studies investigated patients in the late second or third trimester. Very few studies have stratified the adverse outcomes of patients according to the gestational age at which infection occurred (*12*). The objective of our study was to measure the prevalence of obstetric and neonatal outcomes in patients infected with SARS-CoV-2 and to examine the effect of gestational age at infection on each outcome.

## Methods

This international multicenter retrospective cohort study, PregOuTCOV, was conducted in 4 university hospitals in Europe that follow similar guidelines and protocols for antenatal and intrapartum care. The study population consisted of all pregnant women with a viable fetus from the 10th WG and a known pregnancy outcome during February 1–November 30, 2020. The exposed group included pregnant patients in whom nasopharyngeal swab specimens tested positive for SARS-CoV-2 by reverse transcription PCR (RT-PCR) during this period; the unexposed group consisted of the remaining cohort of patients followed in the 4 hospitals during the same period. These patients were either not tested (because there was no indication) or tested negative. We excluded multiple pregnancies, patients with ongoing pregnancies and hence no birth outcomes, those with unknown pregnancy outcomes, those with medical or voluntary pregnancy termination, and patients in whom spontaneous abortion occurred before the 10th WG. None of the centers involved in this study performed regular (by month or by trimester) systematic screening of pregnant women by RT-PCR during the study period; all testing was performed on the basis of clinical symptoms or before planned admissions (regular hospital admission or admission for labor and delivery).

The study was approved by the appropriate ethical board for each recruiting center (approval nos. CE2020/206, CEROG 2020-OBST-1104, and IST DIPUSVSP-24-02-217), and informed consent was obtained when required by the relevant local regulations. Clinical data were routinely collected in real time in the patient’s electronic medical records. Data were then extracted retrospectively for the study and merged into a dedicated, secured, and anonymized database based at the coordinating center. A data control was performed before analysis and, if data were inaccurate or missing, the recruiting centers were contacted to correct the identified issues. All relevant local and Europe privacy regulations were respected.

The collated data included maternal age, geographic origin, prepregnancy BMI, parity, smoking status, chronic arterial hypertension, diabetes mellitus type I or II, preexisting pulmonary diseases (such as asthma, tuberculosis, and previous pulmonary embolism [PE]), and preexisting renal or liver diseases (such as renal or hepatic insufficiency, polycystic kidney disease, single kidney, previous nephrectomy, viral hepatitis, and kidney or liver transplant). For SARS-CoV-2–positive patients, we also collected data on date of positive RT-PCR test, gestational age at the time of RT-PCR, reason for performing RT-PCR (symptoms or screening), hospital admission related to SARS-CoV-2 infection, and disease severity according to the National Institutes of Health (*13*). We also recorded the occurrence of SARS-CoV-2 pneumonia, acute respiratory distress syndrome (*14*), invasive ventilation, oxygen support, and extracorporeal membrane oxygenation.

Primary outcomes of the study were a composite adverse obstetric outcome (CAOO) and a composite adverse neonatal outcome (CANO). CAOO was defined as preterm delivery (<37 WG), preeclampsia, eclampsia, HELLP (hemolysis, elevated liver enzymes, low platelet count) syndrome, unscheduled cesarean delivery, deep venous thrombosis (DVT), PE, pregnancy loss at <24 WG, intrauterine fetal demise (>24 WG), or maternal death. CANO was defined as low birthweight (<2,500 g), neonatal intensive care unit (NICU) admission, APGAR score of <7 at 5 minutes of life, respiratory distress, or neonatal death. The criteria for NICU admission were gestational age at birth of <32 WG, birthweight of <1,500 g, signs of respiratory distress, hemodynamic instability, metabolic problems needing central venous access placement and intensive care, perinatal asphyxia defined according to American College of Obstetricians and Gynecologists and American Academy of Pediatrics criteria, and need for exchange-transfusion (*15*). Neonates of SARS-CoV-2–positive mothers were not systematically admitted to the NICU for monitoring for reasons outside these listed criteria. Secondary outcomes of the study included each outcome of the composite variables, as well as delivery at <32 WG, spontaneous delivery at <37 WG, suspected fetal distress (such as fetal bradycardia or recurrent late or variable decelerations on antepartum or intrapartum cardiotocography), cesarean delivery, postpartum hemorrhage (defined as blood loss of >500 mL in normal vaginal delivery and >1,000 mL in cesarean delivery), umbilical artery pH abnormalities, small for gestational age (defined as estimated fetal weight <10th percentile), and large for gestational age (defined as estimated fetal weight >95th percentile) (*16*).

We performed 2 propensity scores on 2 groups: the maternal population (unexposed group [n = 10,532] and exposed group [n = 393]) and the neonatal population (unexposed group [n = 10,370] and exposed group [n = 388]). After we performed 15 multiple imputations of missing data of the original datasets by using the mice package in R software (R Project for Statistical Computing, https://www.r-project.org/), we used the CBPS R package to perform the propensity score, estimating an average treatment effect using covariate balancing and requesting an exact match, which has been shown to be superior to traditional logistic regression approaches and boosted classification and regression trees (*17*). We considered an absolute standardized difference (ASD) of <10%–15% to support the assumption of balance between the groups because it is not affected by sample size, unlike p values, and it can be used to compare the relative balance of variables measured in different units (*18*). We calculated the mean and SD obtained after matching for continuous variables and the percentage for categorical variables. After performing the propensity score, we used the survey R package to perform logistic regressions for the binary outcome variables, which included the treatment group effect, the weight resulting from the matching, and variables present in the propensity score to obtain a doubly robust estimator, which corrected the last remaining possible imbalance between the covariates and produced an unbiased treatment effect (*19*). The survey R package included the Huber-White corrected SEs, which maintained the SEs unbiased even under heterogeneity of the residuals (*20*). Finally, the advantage of a doubly robust estimator is that it needs only 1 of the 2 models (propensity score and logistic regression after the propensity score) to be correctly specified. We used R software version 3.4.3 (to produce the results. Before matching of covariates, a p value of <0.05 was considered statistically significant. Nevertheless, we had to correct for multiple testing with a Bonferroni correction. For the secondary outcomes, 10 comparisons were performed twice; therefore, we divided the p value by the number of comparisons to obtain the p value at which we considered a result significant (0.05/10 = 0.005). All secondary outcomes with a p value <0.005 after matching were therefore considered significant.

We used the coxme R package to estimate Cox proportional hazards models using the center as random effect on a subset of the data, for which the gestational age at the time of SARS-CoV-2 RT-PCR was collected (for maternal population, unexposed group [n = 2,343] and exposed group [n = 393]; for neonatal population, unexposed group [n = 2,308] and exposed group [n = 383]). This subgroup was representative of the study population (Appendix Table 1). One random effect Cox model with a censor at 41 WG was drawn per outcome, including the outcome of interest and the covariates included in the propensity score. A p value <0.05 was considered significant.

## Results

### Baseline Characteristics

**Figure 1:**
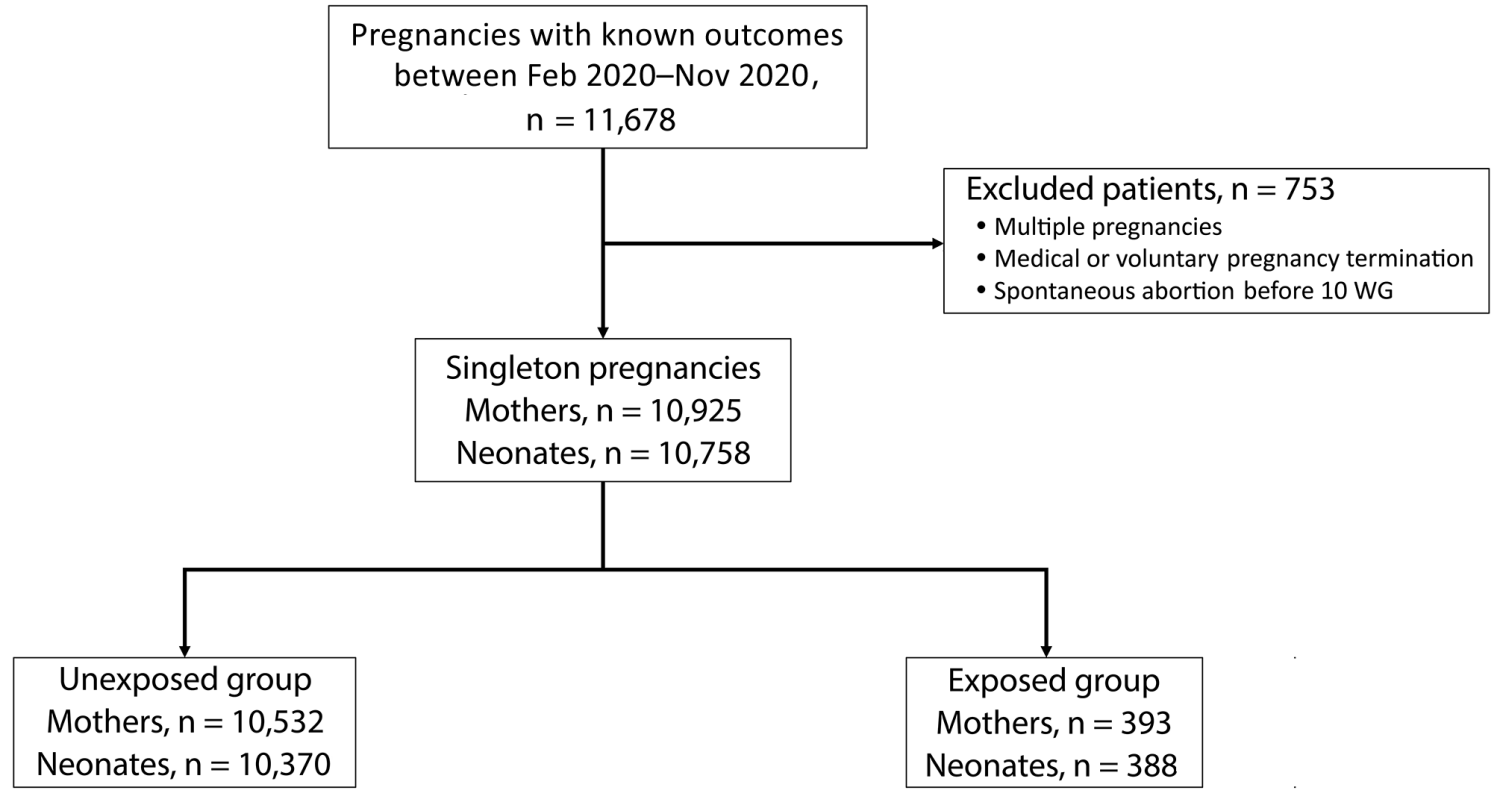
Flowchart of the study population in PregOutCOV study of pregnancy outcomes in Europe according to gestational age at time of infection with severe acute respiratory syndrome coronavirus 2. Pregnancy losses before delivery were excluded from the neonatal population. WG, weeks of gestation.

In total, we identified 10,925 singleton pregnancies that were eligible for final analysis (Table 1). A total of 393 patients tested positive for SARS-CoV-2 (3.60%). Among them, 196 (49.87%) were symptomatic (8 critical, 12 severe, 34 moderate, 135 mild, and 7 not classified). Of these, 46 patients had pneumonia (11.70%) and 16 had acute respiratory distress syndrome (4.07%). A total of 37 patients (9.41%) needed oxygen therapy, whereas 9 (2.29%) needed invasive ventilation. No patients required extracorporeal membrane oxygenation. Among the 10,925 women, 167 had a pregnancy loss and 10,758 delivered a live neonate (Table 2; Figure 1).

**Table 1:** Baseline characteristics before and after covariate matching of 10,925 pregnant women in Europe included in final analysis in PregOutCOV study of pregnancy outcomes according to gestational age at time of infection with severe acute respiratory syndrome coronavirus 2*

Characteristic	Before matching				After matching		
	Unexposed, n = 10,532	Exposed, n = 393	ASD†		Unexposed, n = 10,532	Exposed, n = 393	ASD‡
Mean age, y (SD)	33.05 (+5.43)	33.32 (+5.58)	4.93		33.06 (+5.43)	33.08 (+5.49)	0.34
Origin							
Europe, Middle East, North Africa	78.37	77.35	2.45		78.34	78.39	0.13
Sub-Saharan Africa, Caribbean	13.79	17.56	10.39		13.93	13.94	0.02
Not mentioned by the patient	6.71	3.82	13.00		6.60	6.56	0.14
Not available	1.13	1.27	1.31		1.14	1.10	0.36
Prepregnancy BMI, kg/m^2^ (SD)	25.16 (+5.09)	26.34 (+5.39)	22.50		25.21 (+5.12)	25.24 (+4.96)	0.55
Multiparity	54.12	58.78	9.40		54.26	54.44	0.36
Smoking	11.18	11.20	0.06		11.19	11.13	0.19
Chronic hypertension	1.35	1.27	0.67		1.34	1.33	0.14

*Values are % pregnant women except as indicated. ASD, absolute standardized difference; BMI, body mass index. †ASDs before matching show heterogeneity between the exposed and unexposed groups. ‡ASDs after matching show a balance between the exposed and unexposed groups.

**Table 2:** Baseline characteristics before and after covariate matching of the mothers of the 10,758 live neonates in Europe included in final analysis (after removing 167 patients with pregnancy losses) in PregOutCOV study of pregnancy outcomes according to gestational age at time of infection with severe acute respiratory syndrome coronavirus 2*

Characteristic	Before matching				After matching		
	Unexposed, n = 1,0370	Exposed, n = 388	ASD†		Unexposed, n = 10,370	Exposed, n = 388	ASD‡
Mean age, y (SD)	33.11 (+5.43)	33.31 (+5.61)	3.67		33.11 (+5.42)	33.13 (+5.55)	0.44
Origin							
Europe, Middle East, North Africa	78.52	77.58	2.29		78.49	78.51	0.07
Sub-Saharan Africa, Caribbean	13.63	17.27	10.08		13.76	13.75	0.04
Not mentioned by the patient	6.85	3.87	13.29		6.73	6.76	0.11
Not available	1.00	1.29	2.75		1.02	0.98	0.41
Prepregnancy BMI, kg/m^2^ (SD)	25.65 (+5.99)	26.72 (+5.92)	17.83		25.71 (+6.05)	25.70 (+5.31)	0.11
Multiparity	53.78	59.28	11.10		53.96	53.96	0.01
Smoking	12.10	11.34	2.36		12.10	12.06	0.12
Chronic hypertension	1.26	1.29	0.24		1.26	1.27	0.10

*Values are % pregnant women except as indicated. ASD, absolute standardized difference; BMI, body mass index. †ASDs before matching show heterogeneity between the exposed and unexposed groups. ‡ASDs after matching show a balance between the exposed and unexposed groups.

### Primary Outcomes

The rates of CAOOs and CANOs were significantly higher in SARS-CoV-2–positive patients. CAOOs occurred in 22.75% of exposed persons versus 19.25% of unexposed persons (p<0.001; ASD = 8.62%). CANOs occurred in 17.86% of exposed persons versus 14.28% of the unexposed (p<0.001; ASD = 9.76%) (Tables 3, 4).

**Table 3:** Obstetric outcomes in PregOutCOV study of pregnancy outcomes in Europe according to gestational age at time of infection with severe acute respiratory syndrome coronavirus 2*

Outcome	Unexposed, n = 10,532	Exposed, n = 393	p value	ASD
Primary outcome†				
Composite adverse obstetric outcome	19.25	22.75	<0.001	8.62
Secondary outcome‡				
Preeclampsia, eclampsia, or HELLP syndrome	1.89	2.44	0.004	3.78
Pregnancy loss at <24 weeks	1.06	0.71	0.034	3.73
Pregnancy loss at >24 weeks	1.54	1.19	0.060	2.97
Delivery at <32 weeks	3.18	3.63	0.052	2.51
Delivery at <37 weeks	8.90	12.22	<0.001	10.71
Spontaneous delivery at <37 weeks	5.65	4.96	0.056	2.86
Caesarean delivery	24.68	26.63	0.002	4.17
Unscheduled caesarean delivery	12.27	13.87	<0.001	4.73
Postpartum hemorrhage	9.23	12.57	<0.001	10.74
DVT or PE	0.06	0.53	<0.001	8.77

*Values are % pregnant women except as indicated. ASD, absolute standardized difference; DVT, deep vein thrombosis; HELLP, hemolysis, elevated liver enzymes, low platelet count; PE, pulmonary embolism. †Significant statistical difference: p < 0.05. ‡Significant statistical difference: p < 0.005 (Bonferroni correction).

**Table 4:** Neonatal outcomes in PregOutCOV study of pregnancy outcomes in Europe according to gestational age at time of infection with severe acute respiratory syndrome coronavirus 2*

Outcome	Unexposed, n = 10,370	Exposed, n = 388	p value	ASD
Primary outcome†				
Composite adverse neonatal outcome	14.28	17.86	<0.001	9.76
Secondary outcome†				
Small for gestational age	10.89	9.39	<0.001	4.98
Large for gestational age	6.53	5.60	0.0029	3.87
Fetal distress	8.74	10.95	<0.001	7.44
Neonatal death	0.32	0.14	<0.001	3.66
Birthweight, g (SD)	3228.00 (±579.34)	3128.90 (±602.93)	<0.001	16.76
NICU admission	7.76	13.09	<0.001	17.49
Respiratory distress	7.10	7.86	0.0297	2.89
APGAR <7 at 5 min	2.58	4.01	<0.001	8.03
Umbilical artery pH	7.25 ± 0.08	7.25 ± 0.07	<0.001	11.12

*Values are % pregnant women except as indicated. ASD, absolute standardized difference; NICU, neonatal intensive care unit. †Statistically significant difference: p<0.05. ‡Statistically significant difference: p<0.005 (Bonferroni correction).

### Secondary Outcomes

SARS-CoV-2 infection was associated with an increase of many obstetric and neonatal outcomes, such as preeclampsia, eclampsia, or HELLP syndrome (2.44% vs. 1.89%; p = 0.004, ASD = 3.78%); delivery at <37 weeks (12.22% vs. 8.90%; p<0.001, ASD = 11.71%); cesarean delivery (26.63% vs. 24.68%; p = 0.002, ASD = 4.17%); unscheduled cesarean delivery (13.87% vs. 12.27%; p<0.001, ASD = 4.73%); postpartum hemorrhage (12.57% vs. 9.23%; p<0.001, ASD = 10.74%); DVT or PE (0.53% vs. 0.06%; p<0.001, ASD = 8.77%); fetal distress (10.95% vs. 8.74%; p<0.001, ASD = 7.44%); NICU admission (13.09% vs. 7.76%; p<0.001, ASD = 17.49%); and APGAR of <7 at 5 minutes (4.01% vs. 2.58%; p<0.001, ASD = 8.03%). Neonates in the exposed group also had significantly lower birthweight (mean +SD 3,128.90 g +602.93 g vs. 3,228.00 g +579.34 g; p<0.001, ASD = 16.76%); however, z-scores of birthweight were similar to the unexposed group (Tables 3, 4).

### Effect of Gestational Age at SARS-CoV-2 Infection on Primary and Secondary Outcomes

**Figure 2:**
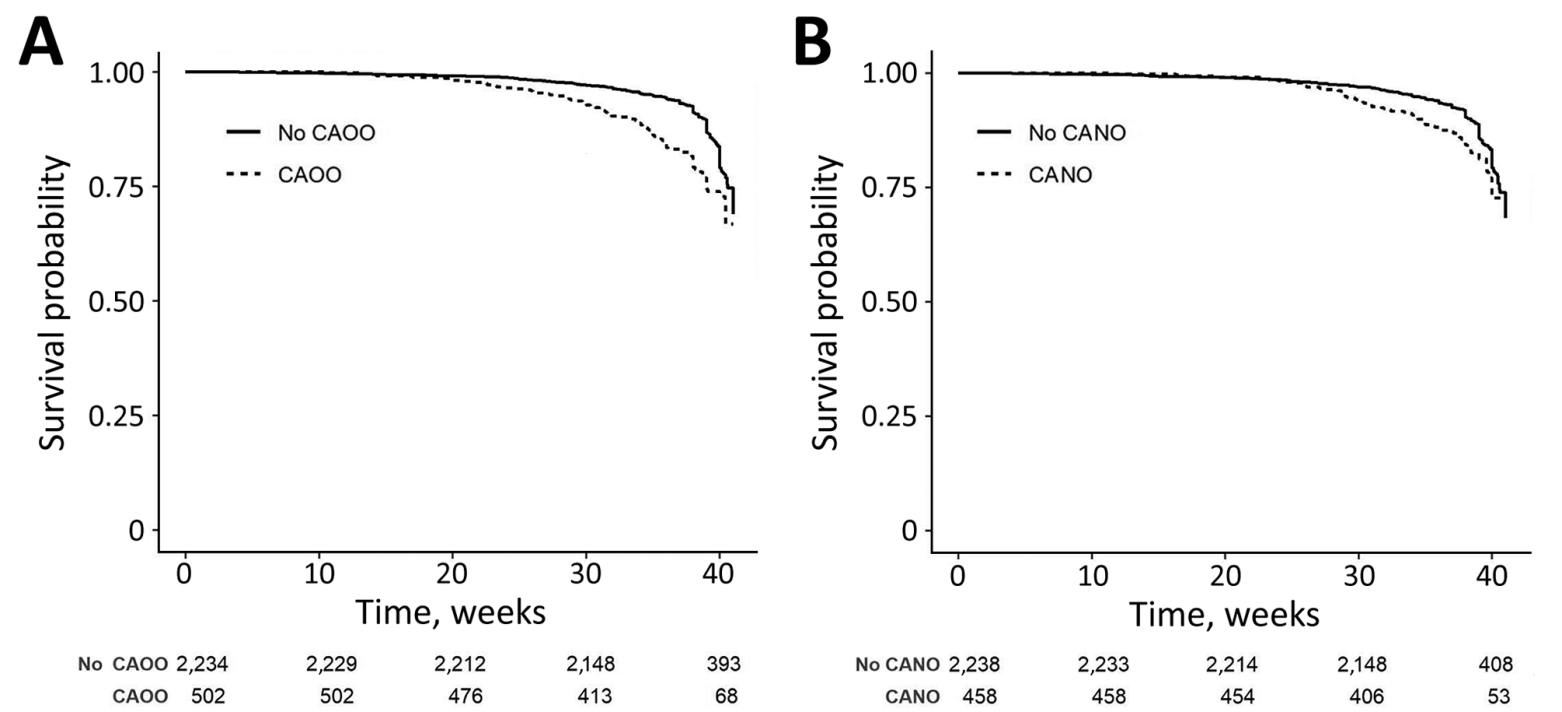
Kaplan-Meier curves demonstrating the effect of gestational age at the time of infection with severe acute respiratory syndrome coronavirus 2 (SARS-CoV-2) on pregnancy outcomes in Europe. A) Compared with patients without CAOO, patients with CAOO were more often infected with SARS-CoV-2. The difference was seen when patients were infected at >20 weeks of gestation. B) Compared with patients without CANO, patients with CANO were more often infected with SARS-CoV-2. The difference was seen when patients were infected at >26 weeks of gestation. Numbers below x-axis indicate number of persons at risk for each time point. CANO, composite adverse neonatal outcome; CAOO, composite adverse obstetric outcome.

Cox regression models demonstrated that patients with CAOOs were more likely to be infected with SARS-CoV-2 than patients without this composite outcome. This difference was seen in patients infected at >20 WG (p<0.001). Similarly, patients with CANOs were more likely to be SARS-CoV-2–positive than patients without this composite outcome. The difference was seen in infected patients at >26 WG (p<0.001) (Figure 2).

Furthermore, when the infection started beyond a defined WG for selected secondary outcomes, the incidence of this outcome increased significantly. These included preeclampsia, eclampsia, or HELLP syndrome (p = 0.002, >15 WG at time of infection); delivery at <37 WG (p<0.001, >24 WG); spontaneous delivery at <37 WG (p<0.001, >26 WG); delivery at <32 WG (p<0.001, >26 WG); NICU admission (p<0.001, >28 WG); and respiratory distress (p<0.001, >28 WG) (Appendix Tables 2, 3, Figures 1, 2).

## Discussion

This study reports the prevalence of adverse obstetric and neonatal outcomes in women infected with SARS-CoV-2 relative to the timing of infection during pregnancy. SARS-CoV-2–positive patients have an increased incidence of adverse obstetric and neonatal outcomes. It appears that pregnant women are more susceptible to the adverse effects of SARS-CoV-2 infection during the late second and early third trimesters.

The effects of SARS-CoV-2 on obstetric and neonatal outcomes has become more evident with time because of the growing body of literature in this area. However, the association between these outcomes and the timing of infection during pregnancy remains unclear. Most studies have reported the obstetric and neonatal outcomes of patients infected in the late second or third trimester. In our study, we included patients who were infected at the beginning of their pregnancies. We demonstrated that gestational age at the time of infection had a critical effect on the incidence of adverse obstetric and neonatal outcomes. SARS-CoV-2 infections after 20 WG significantly increased CAOOs, and infection after 26 WG significantly increased CANOs.

Adverse obstetric and neonatal outcomes, such as preeclampsia, preterm delivery, cesarean delivery, postpartum hemorrhage, and DVT or PE, significantly increased in pregnant women who were infected with SARS-CoV-2. In a meta-analysis published in September 2020 and updated in February 2021 examining 18 studies including 8,549 women, the rate of preterm birth in infected patients was higher than in noninfected patients (odds ratio 1.47, 95% CI 1.14–1.91) (*6*). A systematic review by Wei et al. also confirmed this finding (*21*).

With regard to the incidence of DVT or PE, our findings corresponded to early reports that highlighted the importance of thromboprophylaxis for SARS-CoV-2–positive patients. Most infected hospitalized patients in the 4 institutions in this study received some form of treatment to reduce their risk for DVT and PE (*22*,*23*). Several previous studies have demonstrated an association between preeclampsia and SARS-CoV-2 (*11*,*21*). In a new large observational study, Metz et al. (*24*) grouped 1,219 infected patients according to disease severity. Compared with asymptomatic patients, those with mild to moderate disease had similar rates of cesarean delivery, hypertensive disorders of pregnancy, and preterm birth. Nevertheless, patients with severe to critical disease were at higher risk for these perinatal outcomes. Our study was not designed to compare patients according to disease severity.

Fetal distress during labor, admission of live neonates to the NICU, APGAR scores of <7 at 5 minutes, and umbilical artery pH abnormalities were significantly higher and birthweight was significantly lower in infected patients than in matched unexposed patients. Placental abnormalities, among other factors, might play a role in the occurrence of these elevated risks. Patberg et al. (*25*) compared 77 placentae of infected patients with 56 placentae of noninfected patients and found an increased prevalence of histopathologic abnormalities, such as villitis of unknown etiology and fetal vascular underperfusion, in the SARS-CoV-2–positive group. Shanes et al. reported similar findings (*26*). In addition, Schwartz et al. studied 6 placentae from SARS-CoV-2–positive patients and found that all of them showed chronic histiocytic intervillositis and syncytiotrophoblast necrosis (*27*). These histopathologic abnormalities might interfere with the normal function of the maternal–fetal interface and thereby contribute to the observed adverse neonatal outcomes.

Rates of the remaining outcomes in our study, such as pregnancy loss, neonatal death, small size for gestational age, and large size for gestational age, were either similar or lower in SARS-CoV-2–positive women compared to rates in unexposed pregnant women. In a cohort study of 266 infected pregnant women, Di Mascio et al. estimated pregnancy loss and perinatal death at 6.4% and demonstrated that early gestational age at infection, maternal ventilator supports, and low birthweight were major risk factors for adverse outcomes (*28*). In contrast, in a case-control study of 225 women, Cosma et al. demonstrated that infection during the first trimester might not have a direct effect on spontaneous abortions (*12*). Similarly, a recent study from Denmark found no association between pregnancy loss and SARS-CoV-2 infection during the first trimester (*29*).

Pregnancy is an independent risk factor for respiratory deterioration in patients infected with SARS-CoV-2. Large studies that measure the effect of gestational age at time of infection on obstetric and neonatal outcomes are still lacking. Our study could aid in the counseling of pregnant patients and the organization of antenatal and perinatal care after SARS-CoV-2 infection. Furthermore, this study will help clinicians target pregnant women for SARS-CoV-2 vaccination early enough to provide protection before the crucial threshold of 20 WG. After this gestational age, SARS-CoV-2 infection significantly increases the risk for adverse outcomes.

Prospective studies are needed to examine the effect of the timing of SARS-CoV-2 infection during pregnancy on obstetric and neonatal outcomes. In addition, the possible harmful effects of the virus on placental function, such as chronic histiocytic intervillositis, villitis, and decidual arteriopathy, are still unclear. These placentopathies may be involved in the pathophysiology of adverse obstetric and neonatal outcomes even when the fetus is not directly infected by the virus. More investigations should be targeted at the placental level to learn more about the potentially pathologic and deleterious interactions between the virus and placenta.

This study compares the obstetric and neonatal outcomes of SARS-CoV-2–positive patients according to gestational age at time of infection. Data concerning pregnancy outcomes of patients infected before 20 WG are limited in the current literature. Patients who were infected at the beginning of the pandemic during their first weeks of pregnancy have recently begun to deliver. With this study, we have attempted to address this knowledge gap.

Because it is neither possible nor ethical to expose patients to SARS-CoV-2 infection, the use of propensity score-matching in such situations minimizes selection bias and balances confounding covariates (i.e., age, BMI, parity, and underlying conditions) that could alter between-group differences in obstetric or neonatal outcomes, leaving SARS-CoV-2 infection as the only exposure that could affect these outcomes. Moreover, the inclusion of 4 university hospitals that follow similar guidelines and protocols for antenatal and intrapartum care endorses the findings of this study. None of the recruiting centers used NICU admission to isolate neonates who were born to SARS-CoV-2-positive mothers but had no other need of neonatal critical care. This practice has been discouraged because of a lack of evidence demonstrating a clinical advantage, as well as to avoid unnecessary parent-child separation and NICU bed shortages (*30*).

Nevertheless, these results should be interpreted with caution. The unexposed group will inevitably have included patients who had false-negative SARS-CoV-2 test results or those who were SARS-CoV-2–positive but asymptomatic and not tested. Our chosen methodology means that there will also have been some false-positive results in the infected groups; overall, these small and unavoidable discrepancies would probably have been balanced out by chance. In addition, a single negative result does not exclude an asymptomatic infection that developed later during pregnancy. However, the choice of a contemporaneous unexposed group was the best of the various options available at the time because of various issues relating to the sensitivity, specificity, and the use of the RT-PCR platform in clinical practice (*31*). The exclusion of asymptomatic patients who were not tested for infection also might falsely modify the incidence of obstetric and neonatal outcomes. The selection of an unexposed group before the onset of pandemic might be seen as a reasonable compromise to avoid some of these practical issues. However, this methodology could erroneously modify the incidence of certain outcomes that have also been observed to have changed during the pandemic, even in noninfected women (*7*,*32*).

In conclusion, SARS-CoV-2 infection in pregnant women during the late second and early third trimesters increases the risk for adverse obstetric and neonatal outcomes. However, there is no evidence that infection before 20 WG increases these risks, except for risk for preeclampsia. These findings have implications for public health policy and suggest that vaccination programs should target women either before pregnancy or early in pregnancy to ensure adequate protection when they will be most vulnerable. 

Appendix Additional information about severe acute respiratory syndrome coronavirus 2 and pregnancy outcomes according to gestational age at time of infection.
