# Supplementary material for: Severe Acute Respiratory Syndrome Coronavirus 2 and Pregnancy Outcomes According to Gestational Age at Time of Infection
Source: Emerg Infect Dis. 2021 Oct;27(10):2535–43. doi: 10.3201/eid2710.211394 (PMC8462348; doi:10.3201/eid2710.211394)
Supplement: Appendix — Additional information about severe acute respiratory syndrome coronavirus 2 and pregnancy outcomes according to gestational age at time of infection. [file 21-1394-Techapp-s1.pdf]

Article DOI: https://doi.org/10.3201/eid2710.211394

Severe Acute Respiratory Syndrome Coronavirus 2 and Pregnancy Outcomes According to Gestational Age at Time of Infection

Appendix

Appendix Table 1. Comparison mothers with a gestational age versus those without gestational age within the group not exposed to severe acute respiratory syndrome coronavirus 2*

Variable	Without gestational age	With gestational age
Median age, y (IQR)	33 (29.4–57.74)	33.23 (29.12–37.12)
Diabetes	108/8,172 (1.32)	19/2,336 (0.81)
Hypertension	121/8,172 (1.48)	20/2,336 (0.86)
Asthma	282/8,172 (3.45)	131/2,236 (5.61)
BMI, kg/m^2 (IQR)	24 (21–28.00)	24.98 (22–28.65)

*Values are no. (%) except otherwise indicated. BMI, body mass index.

Appendix Table 2. Cox regression models of secondary obstetric outcomes with survival rates at 41 weeks for the 2 groups in PregOutCOV study of pregnancy outcomes according to gestational age at time of infection with severe acute respiratory syndrome coronavirus 2*

Outcome	Survival rate (95% CI), variable = 0	Survival rate (95% CI), variable = 1	Adjusted p value
Preeclampsia, eclampsia, or HELLP syndrome	68.60% (63.70%–73.90%)	67.00% (49.60%–90.40%)	0.0024
Delivery at <32 weeks	68.80% (63.80%–74.10%)	56.30% (37.70%–84.10%)	<0.001
Delivery at <37 weeks	69.40% (64.40%–74.90%)	41.90% (25.30%–69.40%)	<0.001
Spontaneous delivery at <37 weeks	68.70% (63.70%–74.10%)	45.00% (18.90%–100%)	<0.001
Caesarean delivery	68.70% (63.00%–74.90%)	68.50% (61.10%–76.70%)	0.071
Unscheduled caesarean delivery	68.00% (62.60%–73.90%)	71.40% (62.90%–81.00%)	0.920
Postpartum hemorrhage	68.50% (63.40%–74.10%)	65.90% (48.00%–90.40%)	0.270
DVT or PE	68.50% (63.50%–73.80%)	0.00% (0.00%–0.00%)	0.750

*DVT, deep vein thrombosis; HELLP, hemolysis, elevated liver enzymes, low platelet count; PE, pulmonary embolism.

Appendix Table 3. Cox regression models of secondary neonatal outcomes with survival rates at 41 weeks for the 2 groups in PregOutCOV study of pregnancy outcomes according to gestational age at time of infection with severe acute respiratory syndrome coronavirus 2*

Outcome	Survival rate (95% CI), variable = 0	Survival rate (95% CI), variable = 1	Adjusted p value
Small for gestational age	67.90% (62.50%–73.70%)	74.10 (65.20%–84.30%)	0.120
Large for gestational age	68.20% (63.20%–73.70%)	76.90% (65.30%–90.50%)	0.140
Fetal distress	70.00% (65.10%–75.30%)	52.60% (34.50%–80.30%)	0.890
Neonatal death	68.50% (63.60%–73.80%)	85.70% (63.30%–100%)	0.240
NICU admission	69.00% (64.00%–74.50%)	70.60% (61.00%–81.60%)	<0.001
Respiratory distress	68.20% (63.00%–73.90%)	72.20% (61.70%–84.50%)	<0.001
APGAR <7 at 5 min	68.60% (63.50%–74.00%)	68.70% (54.30%–86.80%)	0.710

*NICU, neonatal intensive care unit.

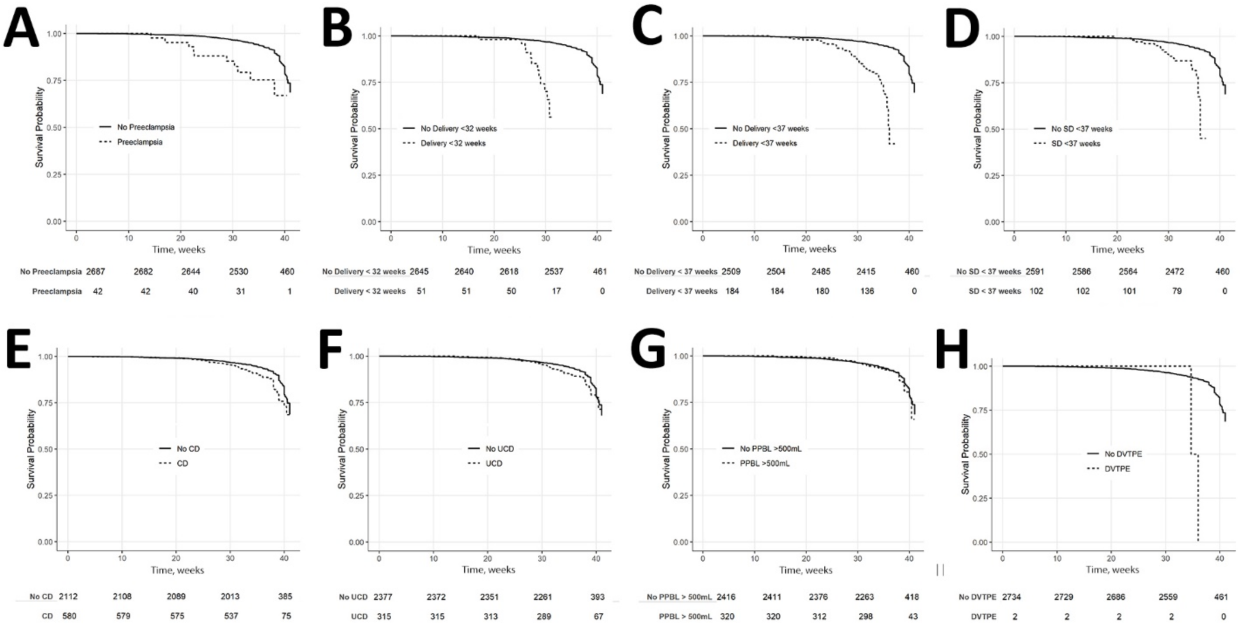

Appendix Figure 1. Kaplan-Meier curves demonstrating the effect of gestational age at the time of severe acute respiratory syndrome coronavirus 2 infection on secondary obstetric outcomes. A) Effect on preeclampsia, B) effect on delivery at <32 weeks, C) effect on delivery at <37 weeks, D) effect on SD at <37 weeks, E) effect on CD, F) effect on UCD, G) effect on PPBL, H) effect on DVTPE. Numbers below x-axis indicate number of persons at risk for each time point. CD, caesarean delivery; DVTPE, deep venous thrombosis or pulmonary embolism; PPBL, postpartum blood loss; SD, spontaneous delivery; UCD, unscheduled caesarean delivery.

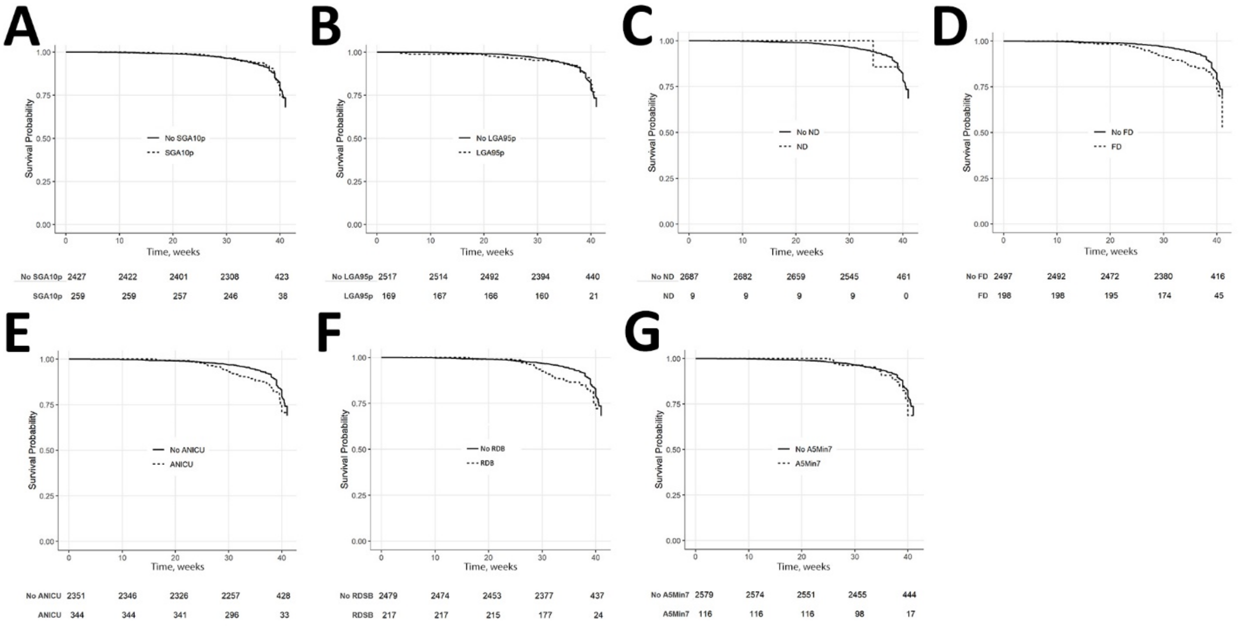

Appendix Figure 2. Kaplan-Meier curves demonstrating the effect of gestational age at the time of severe acute respiratory syndrome coronavirus 2 infection on secondary neonatal outcomes. A) effect on SGA10p, B) effect on LGA95p, C) effect on neonatal death, D) effect on fetal distress, E) effect on ANICU, F) effect on RDB, G) effect on APGAR score at 5 minutes of life. Numbers below x-axis indicate number of persons at risk for each time point. A5Min7, APGAR score of <7 at 5 minutes of life; ANICU, admission to neonatal intensive care unit; LGA95p, large for gestational age (>95th percentile); ND, neonatal death; RDP, respiratory distress at birth; SGA10p, small for gestational age (<10th percentile).
